# Real‐world prevalence of PD‐L1 positivity in early‐stage/metastatic triple‐negative breast cancer: primary results and pathology insights from the global retrospective observational VANESSA study

**DOI:** 10.1111/his.70091

**Published:** 2026-02-22

**Authors:** Corrado D'Arrigo, Sitki Tuzlali, Romualdo Barroso‐Sousa, Nagi S El Saghir, Rebecca Dent, Nataša Medić‐Milijić, Gyungyub Gong, Shahin Sayed, Tu Thai Anh, Alisan Zirtiloglu, Götz Hartleben, Paula Toro, Iman Estaytieh, Enya Weber, Regula Deurloo, João Mouta, Lazar Popovic

**Affiliations:** ^1^ Poundbury Cancer Institute for Personalised Medicine Dorchester UK; ^2^ Tuzlali Pathology Laboratory Istanbul Türkiye; ^3^ Brasília Hospital, Rede Américas Brasilia Brazil; ^4^ American University of Beirut Medical Center Beirut Lebanon; ^5^ National Cancer Center Singapore Duke‐NUS Medical School Singapore Singapore; ^6^ Department of Pathology Institute of Oncology and Radiology of Serbia Belgrade Serbia; ^7^ Department of Pathology, Asan Medical Center University of Ulsan College of Medicine Seoul Republic of Korea; ^8^ Department of Pathology Aga Khan University Nairobi Kenya; ^9^ Pathology Department Ho Chi Minh City Oncology Hospital Ho Chi Minh City Vietnam; ^10^ Global Product Development/Medical Affairs Oncology F. Hoffmann‐La Roche Ltd Basel Switzerland; ^11^ Medical Affairs Roche Pharma AG Grenzach‐Wyhlen Germany; ^12^ Global Medical Affairs Roche Diagnostics International AG Rotkreuz Switzerland; ^13^ Pharmaceuticals Division Roche Lebanon SARL Beirut Lebanon; ^14^ Biometrics and Epidemiology Roche Pharma AG Grenzach‐Wyhlen Germany; ^15^ Translational Medicine Oncology gRED F. Hoffmann‐La Roche Ltd Basel Switzerland; ^16^ Global Product Development/Medical Affairs Oncology Roche Farmacêutica Química Amadora Portugal; ^17^ Department for Medical Oncology, Oncology Institute of Vojvodina, Faculty of Medicine University of Novi Sad Novi Sad Serbia

**Keywords:** PD‐L1, real‐world practice, triple‐negative breast cancer

## Abstract

**Aim:**

To understand whether the worldwide implementation of PD‐L1 testing in triple‐negative breast cancer (TNBC) can be achieved in routine clinical practice.

**Methods and results:**

The multicentre retrospective observational VANESSA study consecutively and uniformly enrolled patients treated with systemic therapy for early or metastatic (e/m)TNBC diagnosed between 2014 and 2017. PD‐L1 status was retrospectively assessed locally and centrally using the VENTANA PD‐L1 (SP142) Assay (PD‐L1 expression on tumour‐infiltrating immune cells covering ≥1% of the tumour area). The primary objective was to determine the prevalence of PD‐L1 positivity assessed locally on primary and/or metastatic tumour tissue. Concordance between local and central testing was a secondary endpoint. PD‐L1‐positive prevalence was 38% in eTNBC (728/1902) and 20% in mTNBC (30/152) and was higher in submitted tissue size >5 versus <5 mm diameter (eTNBC: 43% versus 16%; mTNBC: 24% versus 13%). Among 1967 samples tested both centrally and locally, concordance was 75% (Cohen's κ coefficient 0.52, 95% CI 0.48–0.55) and was similar regardless of cohort (eTNBC versus mTNBC), sample collection method (biopsy versus resection) or sample origin (primary versus metastatic). PD‐L1‐positive prevalence was higher by central versus local assessment (eTNBC: 55% versus 39%; mTNBC: 26% versus 20%).

**Conclusion:**

In this real‐world study, PD‐L1‐positive prevalence was lower than in prospective trials assessing PD‐L1 status centrally, lower in mTNBC than eTNBC, lower in smaller than larger tissue samples and lower by local than central assessment. These findings underline the importance of central PD‐L1 testing on sufficiently large samples to ensure optimal selection for therapies targeting PD‐(L)1 in mTNBC.

## Graphical abstract

In the real‐world VANESSA study in triple‐negative breast cancer, PD‐L1‐positive prevalence was 38% in early‐stage and 20% in metastatic tumours, lower than in prospective trials. The lower prevalence in smaller versus larger tissue samples and by local versus central assessment underlines the importance of robust PD‐L1 testing to determine eligibility for anti‐PD‐(L)1 therapy.

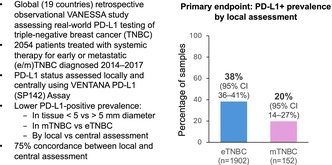

**Abbreviations**
eTNBC: early triple‐negative breast cancer
FFPE: formalin‐fixed paraffin‐embedded
IC: immune cell
mTNBC: metastatic triple‐negative breast cancer
TIL: tumour‐infiltrating lymphocyte
TNBC: triple‐negative breast cancer

## Introduction

In recent years, the management of triple‐negative breast cancer (TNBC), a heterogeneous group of cancers defined by the absence of both hormone receptor expression and overexpression/amplification of HER2, has become increasingly biomarker‐driven. National and international guidelines for TNBC recommend several targeted therapies, including the PARP inhibitors olaparib and talazoparib (for patients with a germline *BRCA1/2* mutation) and the immune checkpoint inhibitors pembrolizumab and atezolizumab (for patients with PD‐L1‐positive advanced disease).^1^, ^2^, ^3^ Consequently, robust and reliable characterization of TNBC, including the presence and nature of gene alterations and the expression of validated biomarkers, has become critical in systemic treatment decision‐making to optimize patient management. Part of this characterization is the accurate determination of PD‐L1 status, which is relevant for locally advanced/metastatic (m)TNBC, but not for early‐stage (e)TNBC. Using the appropriate assay, algorithm and cut‐off value is of paramount importance.

Pivotal phase 3 trials of immune checkpoint inhibitors for both eTNBC and mTNBC have included central PD‐L1 testing using protocol‐defined immunohistochemistry assays and scoring methods, varying according to the investigational agent.^4^, ^5^, ^6^, ^7^, ^8^, ^9^ PD‐L1 scoring using the VENTANA PD‐L1 (SP142) Assay (Roche Diagnostics, Rotkreuz, Switzerland) in TNBC has shown high inter‐ and intra‐observer agreement among trained observers in some studies,^10^, ^11^ but poorer concordance when trained observers test metastatic tissue^12^ or between observers not specifically trained on the assay.^13^ The generalizability of these testing strategies to routine clinical practice at local pathology laboratories is poorly understood. In addition, there is limited information on the prevalence of PD‐L1 positivity outside clinical trials, and it is unclear whether geographical differences in PD‐L1 prevalence exist.

Analyses from IMpassion130 and other studies suggest higher PD‐L1‐positive prevalence in primary tumour compared with metastatic samples, and variation according to metastatic location (lower in liver and soft tissue metastases compared with primary breast lesions or lung or lymph node metastases).^14^, ^15^, ^16^ Temporal changes have also been described, including conversion from negative to positive PD‐L1 status in paired eTNBC and mTNBC samples in the NeoTRIP trial.^17^ Miyakoshi *et al*. reported a particularly pronounced discordance between primary and metastatic samples with the VENTANA PD‐L1 (SP142) Assay.^18^ These variations and heterogeneity highlight the need for a deeper understanding of PD‐L1 testing.

The international VANESSA study assessed the global prevalence of PD‐L1‐positive status in eTNBC and mTNBC using the VENTANA PD‐L1 (SP142) Assay in real‐world clinical practice, including lower‐volume sites testing fewer samples for PD‐L1. This immunohistochemical assay was developed as a companion diagnostic for atezolizumab and is European Community in vitro diagnostic (CE‐IVD) marked for identifying patients with TNBC that is PD‐L1‐positive and suitable for atezolizumab therapy. Here we report the primary results from VANESSA and exploratory analyses relating to diagnostic factors.

## Patients and Methods

### Study Design

The multicentre retrospective observational secondary data‐use VANESSA study enrolled patients treated with systemic therapy for eTNBC/mTNBC. Eligible patients were newly diagnosed with eTNBC or mTNBC (TNBC status assessed locally per ASCO/CAP guidelines^19^, ^20^, ^21^) between 1 January 2014 and 31 December 2017, and with available good‐quality archival formalin‐fixed paraffin‐embedded (FFPE) tumour tissue with documented tissue source, type, size and tumour content. Patients newly diagnosed with mTNBC could have been previously diagnosed with early‐stage breast cancer. Written informed consent was required from all patients (if applicable according to local regulations, or a waiver from the institutional review board/ethics committee if informed consent could not be obtained, e.g. from deceased patients). The study was conducted in full conformance with the Guidelines for Good Pharmacoepidemiology Practice, the laws and regulations of each participating country and the institutional review boards of medical centres.

The target sample size was approximately 2700 patients, balancing feasibility with the precision of estimates in this observational secondary data‐use study. To reduce selection bias, it was planned to enrol eligible patients at each site uniformly and consecutively. High‐volume sites (treating ≥400 newly diagnosed patients with breast cancer annually) enrolled ≥60 patients (e.g. 15 consecutively diagnosed eligible patients from each year) and low‐ to medium‐volume sites (treating <400 newly diagnosed patients with breast cancer annually) enrolled ≥40 patients (e.g. 10 consecutively diagnosed eligible patients from each year). The rationale for restricting enrolment to patients receiving systemic therapy was to study a more homogeneous patient population by excluding those who were not considered to be candidates for systemic therapy because of their very good or poor prognosis.

### Study Objectives

The primary objective was to determine the prevalence of PD‐L1‐positive status assessed locally using the VENTANA PD‐L1 (SP142) Assay on primary and/or metastatic tissue from patients with eTNBC or mTNBC. It was prespecified to explore the primary endpoint in subgroups according to race, sample collection method (biopsy versus surgical resection), tissue size (<5 versus >5 mm, determined externally by the central laboratory), sample origin (primary versus metastatic; mTNBC cohort only) and scoring method (digital versus glass slides).

The secondary objective was to evaluate the concordance between local and central PD‐L1 testing to understand the reproducibility of PD‐L1 testing in real‐world practice. Analysis of concordance within subgroups was prespecified. Analysis of paired samples was planned but is not reported because only small numbers were available.

### Tissue Samples, PD‐L1 Testing and Determination of Tumour‐Infiltrating Lymphocytes (TILs)

The PD‐L1 status of archival tumour tissue samples from primary and/or metastatic lesions was assessed locally and at a central laboratory by pathologists trained and certified in PD‐L1 interpretation specifically for TNBC. PD‐L1‐positive was defined as expression on tumour‐infiltrating immune cells (ICs) covering ≥1% of the tumour area using the VENTANA PD‐L1 (SP142) Assay. IC scores were categorized as IC0 if ICs covered <1%, IC1 for ≥1%–<5% coverage, IC2 for ≥5%–<10% coverage and IC3 for ≥10% coverage.

FFPE tissue block samples had to be sufficient to cut at least eight tissue sections. Slides were cut and stained at the local laboratory for local testing. If eight consecutive slides were not available then two different blocks from the same lesion could be used: one for local and one for central testing. During central testing, staining was repeated on separate slides and scored at the central laboratory. To be eligible, tissue samples were required to have been fixed with 10% buffered formalin for 6–72 h. Staining had to be performed within 60 days of sectioning. Previous local test results could be used if scored by a certified pathologist using the VENTANA PD‐L1 (SP142) Assay. One FFPE tissue block or at least five freshly cut serial sections from the same lesion with an associated pathology report, if available, were required to be submitted for central testing. FFPE tumour tissue samples had to contain ≥50 viable/well‐preserved tumour cells with associated stroma preserving the cellular context and tissue architecture irrespective of the needle gauge or retrieval method. Tumour tissue from bone metastases was not acceptable, nor were samples collected via fine‐needle aspiration, brushing, cell pellets from pleural effusion or lavage samples. TILs were assessed centrally on H&E following International TILs Working Group evaluation guidelines^22^; a ≥10% cut‐off was used for positivity.^14^ Medical and demographic data for exploratory endpoints were retrospectively extracted from each eligible patient's medical records, where available, and recorded in an electronic case report form.

### Statistical Analysis

All analyses were done separately in the eTNBC and mTNBC cohorts and, where possible, by country. Statistical analyses and all comparisons between patient groups and laboratories are descriptive, and no hypothesis testing was performed. Concordance was analysed using Cohen's kappa statistic. The database was locked on 6 June 2024.

## Results

### Patient Population

**Figure 1:**
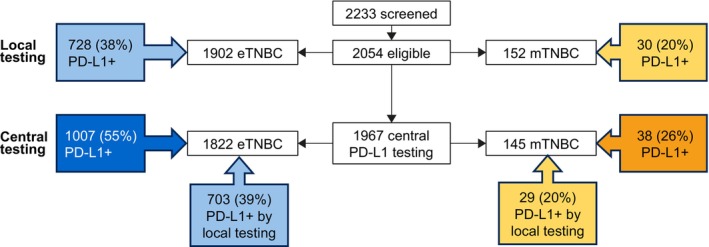
Analysis populations and PD‐L1+ prevalence. eTNBC, early triple‐negative breast cancer; mTNBC, metastatic triple‐negative breast cancer; PD‐L1+, PD‐L1 positive.

Overall, 2054 eligible patients were enrolled from 39 sites (Table S1) in 19 countries across four continents: 1902 with eTNBC and 152 with mTNBC (Figure 1). In the eTNBC cohort, the median age at confirmed histopathological diagnosis was 52 years in both the PD‐L1‐positive and PD‐L1‐negative subgroups (per central assessment; range = 21−98 years); 41% self‐reported as White and 22% as Asian (Table S2). In the mTNBC cohort, the median age was 61 (range = 31–82) years in 38 patients with PD‐L1‐positive mTNBC and 56 (range = 24–89) years in 107 patients with PD‐L1‐negative mTNBC (per central assessment; range = 24–89 years); 52% self‐reported as White and 15% as Asian (Table S2). Only two patients (one with eTNBC, one with mTNBC) were male. In the eTNBC cohort, 40% of patients had stage II disease at diagnosis and 29% had stage III. In the mTNBC cohort, most patients (120/145 [83%] with centrally assessed PD‐L1 status) were diagnosed with de novo metastatic disease.

### PD‐L1‐Positive Prevalence

**Figure 2:**
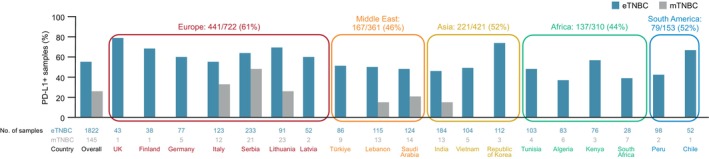
PD‐L1+ prevalence by country (central assessment). eTNBC, early triple‐negative breast cancer; mTNBC, metastatic triple‐negative breast cancer; PD‐L1+, PD‐L1 positive.

The PD‐L1‐positive prevalence by local assessment was 38% (95% CI = 36%–41%) in the eTNBC cohort and 20% (95% CI = 14%–27%) in the mTNBC cohort. There was little difference in PD‐L1‐positive prevalence according to race in the eTNBC cohort (41% [95% CI = 38%–45%] in 778 White patients, 41% [95% CI = 36%–46%] in 423 Asian patients, 30% [95% CI = 21%–40%] in 97 American Indian/Alaska Native patients and 42% [95% CI = 31%–53%] in 89 Black/African American patients). Sample sizes in the mTNBC cohort were too small for meaningful subgroup analysis. PD‐L1‐positive prevalence (by central assessment) varied widely among countries and was higher in most European countries than in other regions, with the notable exceptions of the Republic of Korea, Chile and Kenya (Figure 2).

PD‐L1‐positive status by local assessment was more common in resection material than biopsies in both the eTNBC cohort (46% versus 19%, respectively) and the mTNBC cohort (28% versus 14%, respectively) (Table 1). PD‐L1‐positive status was also more common in larger (>5 mm diameter) than smaller (<5 mm diameter) samples in both the eTNBC cohort (43% versus 16%, respectively) and the mTNBC cohort (24% versus 13%, respectively). In the mTNBC cohort, PD‐L1‐positive prevalence was 19% (95% CI = 12%–27%) in 116 primary tissue samples and 23% (95% CI = 10%–40%) in 35 metastatic samples.

**Table 1:** Prevalence of PD‐L1‐positive status by local versus central assessment and by sample type

Assessment	Subgroup	PD‐L1‐positive samples, *n*/*N* (%) [95% CI]	
		eTNBC	mTNBC
Local (*n* = 2054)	*All patients*	728/1902 (38)	30/152 (20)
	Biopsy (*n* = 619)	99/528 (19) [16–22]	13/91 (14) [8–23]
	Resection (*n* = 1435)	629/1374 (46) [43–49]	17/61 (28) [17–41]
	<5 mm (*n* = 364)^a^	49/312 (16) [12–20]	7/52 (13) [6–26]
	>5 mm (*n* = 1646)^a^	664/1553 (43) [40–45]	22/93 (24) [16–34]
	Light microscopy (*n* = 1933)	697/1796 (39) [37–41]	26/137 (19) [13–27]
	Digital slide (*n* = 121)	31/106 (29) [21–39]	4/15 (27) [8–55]
Central (*n* = 1967)	*All patients*	1007/1822 (55)	38/145 (26)
	IC1	641/1822 (35)	29/145 (20)
	IC2	245/1822 (13)	9/145 (6)
	IC3	121/1822 (7)	0

IC1 = ≥1%–<5% area covered with PD‐L1‐positive ICs as a percentage of the tumour area. IC2 = ≥5%–<10% area covered with PD‐L1‐positive ICs as a percentage of the tumour area. IC3 = ≥10% area covered with PD‐L1‐positive ICs as a percentage of the tumour area.

CI, Confidence interval; eTNBC, Early triple‐negative breast cancer; IC, Immune cell; mTNBC, metastatic triple‐negative breast cancer.

^a^ Subgroup analysis is based on 1865 patients with eTNBC and 145 with mTNBC whose samples had information on tumour size available from the central laboratory.

### Concordance Between Central and Local Assessment

Among 1967 samples (96%) tested both centrally and locally, the PD‐L1‐positive prevalence was higher by central assessment than local assessment in both the eTNBC cohort (55% versus 39%, respectively) and the mTNBC cohort (26% versus 20%, respectively) (Figure 1).

In the overall population combining eTNBC and mTNBC, there was 75% overall percentage agreement between local and central laboratories, represented by 62% positive percentage agreement and 91% negative percentage agreement (Table 2). Concordance was similar in the eTNBC and mTNBC cohorts, regardless of sample collection method (biopsy versus resection) or origin (primary versus metastatic), but lower in samples scored with digital slides versus light microscopy. Table 3 shows concordance by country.

**Table 2:** Concordance between central and local assessment by sample type

Sample type	Percentage agreement, *n*/*N* (%)			Cohen's κ coefficient (95% CI)
	Overall	Positive	Negative	
All patients	1482/1967 (75)	646/1045 (62)	836/922 (91)	0.52 (0.48–0.55)
eTNBC	1362/1822 (75)	625/1007 (62)	737/815 (90)	0.51 (0.47–0.54)
mTNBC	120/145 (83)	21/38 (55)	99/107 (93)	0.52 (0.35–0.68)
Biopsy	477/600 (80)	84/182 (46)	393/418 (94)	0.45 (0.37–0.53)
Resection	1005/1367 (74)	562/863 (65)	443/504 (88)	0.48 (0.44–0.53)
Primary tumour	1429/1895 (75)	625/1009 (62)	804/886 (91)	0.52 (0.48–0.55)
Metastatic site	53/72 (74)	21/36 (58)	32/36 (89)	0.47 (0.28–0.67)
Digital slide	73/116 (63)	28/66 (42)	45/50 (90)	0.30 (0.16–0.44)
Light microscopy	1409/1851 (76)	618/979 (63)	791/872 (91)	0.53 (0.49–0.57)

CI, Confidence interval; eTNBC, Early triple‐negative breast cancer; mTNBC, metastatic triple‐negative breast cancer.

**Table 3:** Concordance between central and local assessment by country

Country	eTNBC			mTNBC		
	PD‐L1 positive (central assessment), *n*/*N* (%)	Overall percentage agreement, *n*/*N* (%)	Cohen's κ coefficient (95% CI)	PD‐L1 positive (central assessment), *n*/*N* (%)	Overall percentage agreement, *n*/*N* (%)	Cohen's κ coefficient (95% CI)
All patients	1007/1822 (55)	1362/1822 (75)	0.51 (0.47–0.54)	38/145 (26)	120/145 (83)	0.52 (0.35–0.68)
Algeria (*n* = 89)	31/83 (37)	58/83 (70)	0.25 (0.07–0.44)	2/6 (33)	4/6 (67)	
Chile (*n* = 53)	35/52 (67)	37/52 (71)	0.45 (0.25–0.66)	1/1 (100)	0/1 (0)	
Finland (*n* = 39)	26/38 (68)	30/38 (79)	0.53 (0.25–0.82)	1/1 (100)	1/1 (100)	
Germany (*n* = 82)	46/77 (60)	61/77 (79)	0.59 (0.42–0.76)	2/5 (40)	4/5 (80)	0.55 (−0.16 to 1.00)
India (*n* = 197)	85/184 (46)	156/184 (85)	0.69 (0.59–0.80)	2/13 (15)	12/13 (92)	0.76 (0.31–1.00)
Italy (*n* = 135)	68/123 (55)	96/123 (78)	0.55 (0.41–0.70)	4/12 (33)	10/12 (83)	0.63 (0.16–1.00)
Kenya (*n* = 79)	43/76 (57)	57/76 (75)	0.51 (0.32–0.69)	0/3 (0)	3/3 (100)	
Latvia (*n* = 54)	31/52 (60)	41/52 (79)	0.59 (0.39–0.79)	0/2 (0)	2/2 (100)	
Lebanon (*n* = 128)	57/115 (50)	81/115 (70)	0.41 (0.27–0.54)	2/13 (15)	11/13 (85)	
Lithuania (*n* = 114)	63/91 (69)	59/91 (65)	0.32 (0.15–0.49)	6/23 (26)	18/23 (78)	0.47 (0.07–0.86)
Peru (*n* = 100)	42/98 (43)	71/98 (72)	0.42 (0.24–0.59)	1/2 (50)	1/2 (50)	
Saudi Arabia (*n* = 138)	59/124 (48)	87/124 (70)	0.39 (0.25–0.53)	3/14 (21)	12/14 (86)	0.58 (0.05–1.00)
Serbia (*n* = 254)	149/233 (64)	186/233 (80)	0.60 (0.50–0.70)	10/21 (48)	17/21 (81)	0.62 (0.28–0.95)
South Africa (*n* = 35)	11/28 (39)	21/28 (75)	0.43 (0.10–0.76)	0/7 (0)	7/7 (100)	
Republic of Korea (*n* = 115)	83/112 (74)	87/112 (78)	0.55 (0.41–0.69)	0/3 (0)	3/3 (100)	
Tunisia (*n* = 107)	49/103 (48)	71/103 (69)	0.37 (0.20–0.54)	1/4 (25)	2/4 (50)	−0.33 (−0.77 to 0.10)
Türkiye (*n* = 95)	44/86 (51)	70/86 (81)	0.63 (0.47–0.79)	2/9 (22)	8/9 (89)	0.61 (−0.06 to 1.00)
UK (*n* = 44)	34/43 (79)	21/43 (49)	0.15 (−0.02 to 0.32)	1/1 (100)	0/1 (0)	
Vietnam (*n* = 109)	51/104 (49)	72/104 (69)	0.38 (0.22–0.54)	0/5 (0)	5/5 (100)	

CI, Confidence interval; eTNBC, Early triple‐negative breast cancer; mTNBC, metastatic triple‐negative breast cancer.

Exploratory analyses revealed that almost half (286/622; 46%) of the discordant eTNBC samples were classified as IC0 locally but IC1 centrally (Table 4). An additional 36/1008 (4%) samples classified as IC0 by local assessment were IC2 or IC3 by central assessment. Conversely, of 691 samples classified as IC0 by central assessment, 49 (7%) were classified locally as IC1–IC3. In the mTNBC cohort, 15/100 samples (15%) classified as IC0 by local assessment were IC1 (13%) or IC2 (2%) by central assessment; 6 (7%) of 86 samples classified as IC0 by central assessment were classified as IC1 or IC2 by local assessment (none as IC3).

**Table 4:** Concordance between central and local assessment by PD‐L1 status. (A) eTNBC cohort (*n* = 1600 samples with locally assessed IC category available). (B) mTNBC cohort (*n* = 125 samples with locally assessed IC category available). Green shading represents concordant results.

Central assessment, *n* (%)	Local assessment, *n* (%)			
	IC0 (*n* = 1008)	IC1 (*n* = 272)	IC2 (*n* = 163)	IC3 (*n* = 157)
**(A) eTNBC**				
IC0	642 (64)	29 (11)	11 (7)	9 (6)
IC1	286 (28)	155 (57)	70 (43)	20 (12)
IC2	29 (3)	68 (25)	50 (31)	61 (39)
IC3	7 (1)	7 (3)	25 (15)	65 (41)
Missing	44 (4)	13 (5)	7 (4)	2 (1)

Central assessment, *n* (%)	Local assessment, *n* (%)			
	IC0 (*n* = 100)	IC1 (*n* = 12)	IC2 (*n* = 6)	IC3 (*n* = 7)
**(B) mTNBC**				
IC0	80 (80)	4 (33)	2 (33)	0
IC1	13 (13)	7 (58)	4 (67)	2 (29)
IC2	2 (2)	0	0	5 (71)
IC3	0	0	0	0
Missing	5 (5)	1 (8)	0	0

eTNBC, Early triple‐negative breast cancer; IC, Immune cell; mTNBC, metastatic triple‐negative breast cancer.

### PD‐L1 Membrane Staining in Tumour Cells and TILs

**Figure 3:**
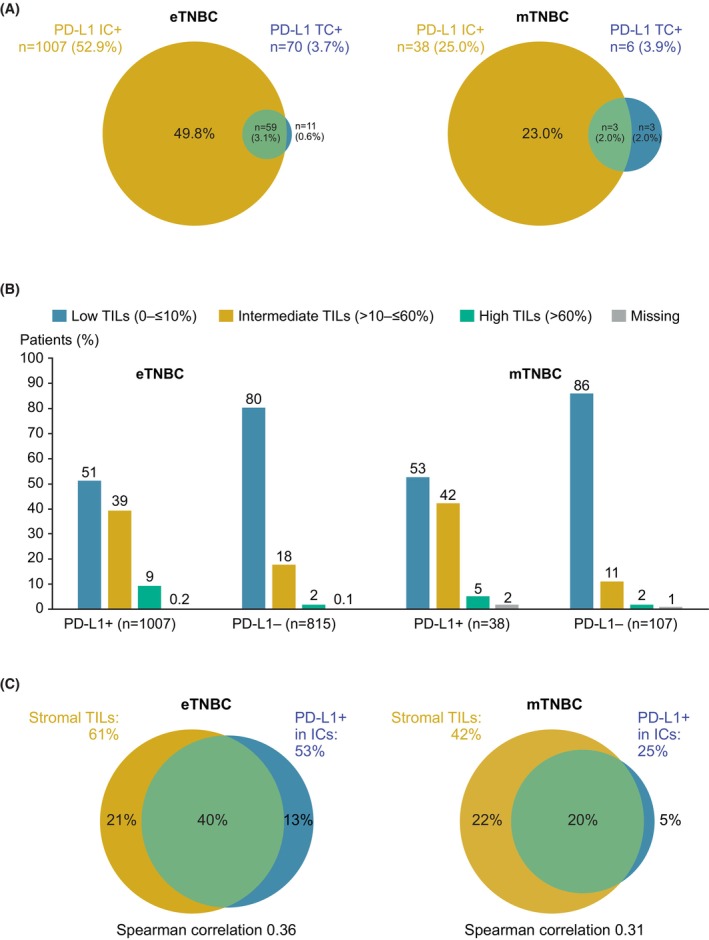
Relationship between PD‐L1 status in ICs and TCs and stromal TILs. (**A**) PD‐L1 status in ICs and TCs. (**B**) Stromal TIL status according to PD‐L1 status (central assessment). (**C**) Association between TILs and PD‐L1 IC positivity (central assessment). eTNBC, early triple‐negative breast cancer; IC, immune cell; mTNBC, metastatic triple‐negative breast cancer; PD‐L1+, PD‐L1 positive; PD‐L1–, PD‐L1 negative; TC, tumour cell; TIL, tumour‐infiltrating lymphocyte.

Among 1902 eTNBC samples, ≥1% membrane staining of tumour cells per central assessment was observed in 70 patients (3.7%), broadly overlapping with PD‐L1‐positive IC (Figure 3A). In the mTNBC cohort, six of 152 samples (3.9%) had ≥1% membrane staining of tumour cells, partially overlapping with PD‐L1‐positive ICs.

Figure 3B shows stromal TILs status by central assessment according to PD‐L1 status. In both the eTNBC and the mTNBC cohorts, the stromal TILs category tended to be lower in PD‐L1‐negative than PD‐L1‐positive samples. Centrally assessed PD‐L1‐positive status in ICs overlapped broadly with stromal TILs (Figure 3C).

## Discussion

In the real‐world VANESSA study, the PD‐L1‐positive prevalence assessed using the VENTANA PD‐L1 (SP142) Assay was lower in mTNBC than in eTNBC (20% versus 38%, respectively), in smaller samples than in larger samples and by local assessment than by central assessment. When comparing centrally assessed PD‐L1‐positive prevalence in VANESSA with data from prospective randomized phase 3 clinical trials, we observed a considerably lower prevalence of PD‐L1 positivity in mTNBC in the present study. In VANESSA, 26% of centrally assessed mTNBC samples were PD‐L1‐positive compared with 41%–45% in IMpassion130^4^ and IMpassion131,^5^ 39% in a recent US real‐world data study,^23^ and 38% in smaller studies from Malaysia^24^ and Korea.^25^ While we cannot exclude that this is related to the relatively small cohort of only 145 patients with mTNBC, one potential explanation for the lower PD‐L1‐positive prevalence in VANESSA could be the testing of archival tissue. In the eTNBC cohort of VANESSA, the PD‐L1‐positive prevalence by central assessment was 55%, compared with 46% in IMpassion031,^7^ 36% in NSABP B‐59/GBG‐96‐GeparDouze^26^ (both in the preoperative setting) and 71% in IMpassion030^27^ (in the postoperative setting). Reports in the literature range from 29% to 53% in series of Asian patients^28^, ^29^, ^30^ and from 46% to 62% in series of US patients.^31^, ^32^

We observed a higher PD‐L1‐positive prevalence in larger than in smaller diameter samples in both eTNBC and mTNBC. This finding is probably related to the often heterogeneous distribution of IC infiltration within TNBC that increases the likelihood of IC‐poor areas in small samples, as opposed to tissue from resection specimens that provide a more representative sample of the tumour microenvironment. Bias in small samples is well documented^33^ and some routine clinical practices repeat PD‐L1 testing when small samples are negative. This was not possible in the retrospective VANESSA study due to limited sample availability.

The VANESSA study found geographic variations in the prevalence of PD‐L1‐positive TNBC. While differences in sample sizes and in processing and storing tissue may have contributed to this geographic variability, true differences in tumour biology and host immune responses cannot be ruled out. PD‐L1‐positive prevalence was lower in samples from biopsies versus surgical resections, consistent with previous reports using the SP142 assay. Noske *et al*. reported poor concordance between preoperative biopsies and surgical specimens^34^ and, in a study of 73 matched core biopsy and excision samples, Dobritoiu *et al*. reported a good correlation in PD‐L1‐positive cases but considerable discordance in PD‐L1‐negative cases.^33^

Previous studies have reported a higher prevalence of PD‐L1‐positive status in primary tumour tissue compared with metastatic tissue.^14^, ^32^ This difference is unsurprising, as the metastatic environment is immune‐deprived, with fewer ICs (including TILs) as cancer cells show more immune‐evasive biology.^35^, ^36^ In VANESSA, the prevalence in mTNBC was lower than in eTNBC, but these represent different cohorts of patients. The demonstration of a difference in PD‐L1 expression between eTNBC and mTNBC samples should ideally rely on the analysis of paired samples. Although this was allowed and encouraged in VANESSA, ultimately very few paired samples were submitted. In analyses of samples from the VANESSA mTNBC cohort, we observed similar PD‐L1‐positive prevalence in primary and metastatic tumour samples (19% and 23%, respectively), but the availability of only 35 metastatic samples precludes any definitive conclusions from this dataset. Likewise, PD‐L1‐positive prevalence by metastatic site cannot be interpreted from such a small dataset.

In both mTNBC and eTNBC, PD‐L1‐positive prevalence was higher by central assessment than local assessment. This discrepancy may be explained by the differing analytical performance of central versus local laboratories, differences between the tissue stained centrally (which was re‐cut and shipped, both of which can influence the analytical performance of the tissue) or differences in interpretation. Future work should assess the relative contribution of these variables, and if differences in interpretation are significant, training programmes should be designed to minimize this variation. Further analyses indicated that most discordant cases were classified as IC0 by local testing but IC1 by central testing. This observation, which may have significant implications for the eligibility of patients for immunotherapy, supports previous studies that have highlighted the difficulty in assessing low‐expressing samples.^37^, ^38^ In these cases, it has been suggested that optimizing preanalytical and analytical procedures, testing a different sample and/or referring for expert opinions may be beneficial.^37^ Interestingly, the International Immuno‐Oncology Biomarker Working Group reported greater discordance in PD‐L1 assessment using SP142 in TNBC metastases than in eTNBC,^12^ suggesting that the focus of training should be on metastatic specimens. Additional training on mTNBC specimens may be particularly appropriate because, based on currently licensed indications and available therapies, PD‐L1 status may influence treatment selection in mTNBC and not in eTNBC.

We found no evidence of greater discordance between local and central assessment in biopsy versus surgical resection samples, or in primary versus metastatic samples. There was poor concordance between local and central assessments of samples scored from digital slides, although it is difficult to draw conclusions based on 116 samples. Given the increasing use of digital pathology in clinical practice, and its relevance particularly in remote areas with limited access to high‐volume pathology centres, there is a clear need for more research in this field to understand the reliability and reproducibility of digital scoring. Implementing standardization processes for digital PD‐L1 assessment and ensuring a minimum level of reproducibility is critical in scaling up this important resource.

The higher stromal TILs status in PD‐L1‐positive samples than PD‐L1‐negative samples is consistent with previous reports,^28^ and could contribute to a better response to immunotherapy.^39^ Furthermore, TILs are prognostic for disease‐free survival in TNBC.^40^ The overlap between PD‐L1‐positive status in ICs and stromal TILs is consistent with recent reports in the literature.^41^

To the best of our knowledge, this is the first study to explore the prevalence of PD‐L1 positivity in an unselected global population of patients presenting with TNBC in real‐world practice. Extensive efforts were made to reduce selection bias through consecutive and uniform enrolment; however, the exceptionally high percentage of patients with de novo mTNBC in this study is surprising and may reflect some degree of enrolment bias, limiting interpretation and the applicability of findings from the mTNBC cohort to a broader population. In addition, the enrolment of a population dominated by eTNBC limits generalizability to mTNBC. Acknowledging the well‐recognized differences between assays,^42^, ^43^ these results using the VENTANA PD‐L1 (SP142) Assay should not be extrapolated to all PD‐L1 assays. Furthermore, the assay used does not provide qualitative information on the composition of the ICs. Another limitation is the lack of paired samples, preventing detailed study of intra‐patient changes in PD‐L1 status through the disease course and under treatment pressure. The heterogeneity of clinical practice among participating countries (including diagnostic and staging procedures, treatment approaches, standard practice for follow‐up and the quality, completeness and format of medical records) also challenges interpretation.

Correctly determining PD‐L1 status is very important in patients with mTNBC because of the therapeutic implications. In clinical practice, some centres may use the combined positive score in patients whose tumours show a VENTANA PD‐L1 (SP142) Assay score <1%—and, conversely, SP142 scoring if the combined positive score is <10—to maximize the likelihood of eligibility for immunotherapy (atezolizumab or pembrolizumab).^44^ Training efforts may help to ensure patients with PD‐L1‐positive tumours are not missed by local testing, thereby maximizing the chances of improved clinical outcomes.

## Conclusion

Results from the VANESSA study highlight the complexity of PD‐L1 assessment and the importance of testing on adequately sized tissue samples. Efforts to establish regional or in‐country reference testing centres are critical to ensure optimal selection for therapies targeting PD‐(L)1 in patients with mTNBC. Even with formal training, there may still be variability in testing protocols, including sample collection, processing, storage, retrieval, re‐processing and testing. Continued efforts should be made to improve the quality and inter‐laboratory concordance of PD‐L1 status assessment across geographic regions, in which leveraging emerging technologies, such as AI algorithms, may play a role.

## Funding information

This study was sponsored and funded by F. Hoffmann‐La Roche Ltd.

## Conflict of interest

C D'Arrigo is the founder of the Poundbury Cancer Institute and has received personal funds for consultation and advisory roles as well as funds for research projects and studies from Roche, AstraZeneca, Daiichi Sankyo Inc., Merck Sharp & Dohme and Pfizer. R Barroso‐Sousa reports receiving speaker bureau fees from AstraZeneca, Daiichi Sankyo, Eli Lilly, Pfizer, Novartis, Merck and Roche, has served as a consultant/advisor for AstraZeneca, Eli Lilly, Libbs, Roche and Merck, has received support for attending medical conferences from AstraZeneca, Roche, Eli Lilly, Daiichi Sankyo and Merck, has received institutional research funding from AstraZeneca and Daiichi Sankyo, and owns stocks in the educational company RD Educacao Medica LTDA. N S El Saghir reports honoraria for lectures from AstraZeneca, Novartis and Roche. R Dent reports consulting fees/honoraria/advisory boards/travel expenses from AstraZeneca, MSD, Pfizer, Eisai, Novartis, Daiichi Sankyo, Roche, Eli Lilly & Company, Genentech and Gilead and research grants from AstraZeneca and Roche. A Zirtiloglu, G Hartleben, P Toro, I Estaytieh, E Weber, R Deurloo and J Mouta are current or former employees of and shareholders in Roche. L Popovic reports speaker/advisor/investigator roles for AstraZeneca, MSD, BMS, Pfizer, Roche, Merck, Novartis, Lilly, Gilead, Takeda, Helsinn, Astellas, Janssen, Sanofi, Sandoz, Actavis, Amgen, Archigen, Amicus, Taiho, Infinity, Bioclin, G1 Therapeutics, MEI Pharma, Immunocore/Medison, NAPO Pharmaceuticals, Oktal, PharmaSwiss, AbbVie, MedicaLinea, MAK pharma, Agendia, Recordati, Incyte and Bicycle Therapeutics. S Tuzlali, N Medić‐Milijić, G Gong, S Sayed and AT Thai declare no conflicts of interest.

## Patient consent statement

Written informed consent was required from all patients (if applicable according to local regulations, or a waiver from the institutional review board/ethics committee if informed consent could not be obtained, for example from deceased patients).

## Supporting information


**Table S1.** Participating sites and principal physicians. Three sites enrolled patients subsequently excluded from the eligible patient population.
**Table S2.** Self‐reported race.

## Data Availability

Data from which the published results of the VANESSA study are derived are archived by the study sponsor and can be made publicly available upon request. Requests for the data underlying this publication require a detailed, hypothesis‐driven statistical analysis plan that is collaboratively developed by the requestor and company subject matter experts. Direct such requests to joao.mouta@roche.com for consideration.
