# Supplementary material for: Real‐world prevalence of PD‐L1 positivity in early‐stage/metastatic triple‐negative breast cancer: primary results and pathology insights from the global retrospective observational VANESSA study
Source: Histopathology. 2026 Feb 22;88(7):1373–84. doi: 10.1111/his.70091 (PMC13128325; doi:10.1111/his.70091)
Supplement: Supplementary file 1 — Table S1. Participating sites and principal physicians. Three sites enrolled patients subsequently excluded from the eligible patient population. Table S2. Self‐reported race. [file HIS-88-1373-s001.docx]

**Supplementary Table S1.** Participating sites and principal physicians. Three sites enrolled patients subsequently excluded from the eligible patient population.

| Country | Principal physician | Site |
| --- | --- | --- |
| Serbia (*n =* 268) | Nataša Medić-Milijić | Institute of Oncology and Radiology of Serbia, Belgrade |
|  | Ana Cvetanovic | University Clinical Centre Nis, Nis |
|  | Lazar Popovic | Oncology Institute of Vojvodina, Sremska Kamenica |
| India (*n =* 200) | S. Ganapathi Raman | Apollo Hospitals, Anna Salai, Chennai |
|  | Pavithran Keechilat | Amrita Institute of Medical Sciences, Ponekkara |
|  | Dinesh Doval | Rajiv Gandhi Cancer Institute & Research Center, Rohini, New Delhi |
|  | Sudeep Gupta | Tata Memorial Hospital, Parel, Mumbai |
| Italy (*n =* 150) | Filippo Giovanardi | AUSL-IRCCS di Reggio Emilia, Ospedale Civile di Guastalla, Reggio Emilia |
|  | Giulia Bianchi | IRCCS Istituto Nazionale dei Tumori, Milano |
|  | Patrizia Vici | IRCCS Regina Elena National Cancer Institute, Rome |
|  | Claudia Bighin | IRCCS A.O.U San Martino – IST, Genova |
| Saudi Arabia (*n =* 139) | Abdulmohsen Alkushi | National Guard King Abdulaziz Medical City, Riyadh |
|  | Sayed Akhtar | King Faisal Specialist Hospital & Research Centre, Riyadh |
| Lebanon (*n =* 128) | Nagi El Saghir | American University of Beirut – Medical Center, Beirut |
|  | Fadi Farhat | Hammoud Hospital, Saida |
|  | David Attalah | Hotel Dieu de France, Beirut |
| Republic of Korea (*n =* 120) | Gyungyub Gong | Asan Medical Center, Seoul |
| Vietnam (*n =* 120) | Tu Thai Anh | Ho Chi Minh City Oncology Hospital, Ho Chi Minh |
|  | To Ta | K Hospital, Ha Noi |
| Lithuania (*n =* 117) | Elona Juozaityte | Hospital of Lithuanian University of Health Sciences Kaunas Clinics, Kaunas |
|  | Monika Drobniene | National Cancer Institute, Vilnius |
| Tunisia (*n =* 117) | Maha Driss | Salah Azaiez Institute, Tunis |
|  | Moncef Mokni | Farhat Hached Hospital, Sousse |
| Peru (*n =* 100) | Henry Gomez-Moreno | Oncosalud Sac, Lima |
|  | Carlos Castañeda | Instituto Nacional de Enfermedades Neoplasicas, Lima |
| Algeria (*n =* 99) | Amel Ladjeroud and Mohammed Oukkal | CPMC, Alger, Algiers (satellite site: CHU Béni-Messous, Beau-Fraisiers) |
|  | Hanene Djedi | CHU Annaba, Hôpital Dorban, Annaba |
| Türkiye (*n =* 99) | Handan Kaya | Marmara University Faculty of Medicine, Istanbul |
|  | Arsenal Alikanoglu | Antalya Research and Training Hospital, Antalya |
|  | Ebru Tastekin | Trakya University Medical Faculty, Edirne |
|  | Melek Ergin | Cukurova University Medical Faculty, Adana |
| Germany (*n =* 86) | Ingolf Juhasz-Böss | Universitätsklinikum Freiburg, Freiburg |
|  | Eugen Ruckhäberle | Universitätsklinikum Düsseldorf, Düsseldorf |
| Kenya (*n =* 80) | Shahin Sayed | Aga Khan University Hospital, Nairobi |
| Chile (*n =* 56) | Felipe Reyes | Fundacion Arturo Lopez Perez, Santiago |
|  | Cesar Sanchez | Centro de Cancer Pontificie Universidad Catolica de Chile, Santiago |
| Latvia (*n =* 54) | Janis Eglitis | Riga East Clinical University Hospital Latvian Oncology Centre, Riga |
| UK (*n =* 44) | Corrado D’Arrigo | Poundbury Cancer Institute, Dorchester |
| Finland (*n =* 40) | Antti Ellonen | Turku University Central Hospital, Turku |
| South Africa (*n =* 37) | Georgia Savva Demetriou | Charlotte Maxeke Johannesburg Academic Hospital, Johannesburg |
| Morocco (*n =* 0) | Nawfel Mellas | Centre Hospitalier Universitaire Hassan II |
|  | Hassan Errihani | Institut National d’Oncologie Sidi Mohammed Ben Abdellah |

**Supplementary Table S2.** Self-reported race

| Self-reported race, *n* (%) | eTNBC (*n* = 1902) | mTNBC (*n* = 152) |
| --- | --- | --- |
| American Indian or Alaska Native | 97 (5) | 1 (1) |
| Asian | 423 (22) | 23 (15) |
| Black or African American | 89 (5) | 6 (4) |
| Native Hawaiian or other Pacific Islander | 2 (0.1) | 0 |
| White | 778 (41) | 79 (52) |
| Other | 225 (12) | 18 (12) |
| Unknown/missing | 288 (15) | 25 (16) |
